# Acute physiological effects of glucocorticoids on fuel metabolism in humans are permissive but not direct

**DOI:** 10.1111/dom.12899

**Published:** 2017-03-28

**Authors:** Roland H. Stimson, Anna J. Anderson, Lynne E. Ramage, David P. Macfarlane, Andrew C. de Beaux, Damian J. Mole, Ruth Andrew, Brian R. Walker

**Affiliations:** ^1^ Centre for Cardiovascular Science, Queen's Medical Research InstituteUniversity of EdinburghEdinburghUK; ^2^ Department of Upper GI SurgeryRoyal Infirmary of EdinburghEdinburghUK; ^3^ MRC Centre for Inflammation ResearchQueen's Medical Research Institute, University of EdinburghEdinburghUK

**Keywords:** adipose tissue, adrenaline, body composition, drug mechanism, glucocorticoid, glucose, insulin resistance, metabolism

## Abstract

**Background and aims:**

The effects of glucocorticoids on fuel metabolism are complex. Acute glucocorticoid excess promotes lipolysis but chronic glucocorticoid excess causes visceral fat accumulation. We hypothesized that interactions between cortisol and insulin and adrenaline account for these conflicting results. We tested the effect of cortisol on lipolysis and glucose production with and without insulin and adrenaline in humans both in vivo and in vitro.

**Materials and methods:**

A total of 20 healthy men were randomized to low and high insulin groups (both n = 10). Subjects attended on 3 occasions and received low (c. 150 nM), medium (c. 400 nM) or high (c. 1400 nM) cortisol infusion in a randomized crossover design. Deuterated glucose and glycerol were infused intravenously along with a pancreatic clamp (somatostatin with replacement of glucagon, insulin and growth hormone) and adrenaline. Subcutaneous adipose tissue was obtained for analysis. In parallel, the effect of cortisol on lipolysis was tested in paired primary cultures of human subcutaneous and visceral adipocytes.

**Results:**

In vivo, high cortisol increased lipolysis only in the presence of high insulin and/or adrenaline but did not alter glucose kinetics. High cortisol increased adipose mRNA levels of ATGL, HSL and CGI‐58 and suppressed G0S2. In vitro, high cortisol increased lipolysis in the presence of insulin in subcutaneous, but not visceral, adipocytes.

**Conclusions:**

The acute lipolytic effects of cortisol require supraphysiological concentrations, are dependent on insulin and adrenaline and are observed only in subcutaneous adipose tissue. The resistance of visceral adipose tissue to cortisol's lipolytic effects may contribute to the central fat accumulation observed with chronic glucocorticoid excess.

## 1 INTRODUCTION

Glucocorticoids are critical regulators of energy balance; however, their complex effects on fuel metabolism are highly context‐dependent, are not linear in their dose response and are influenced by factors such as the diurnal rhythm.^1^ One area exemplifying this lack of certainty is the effects of glucocorticoids on adipose tissue.^2^ The prevailing belief is that, in times of acute stress, high cortisol concentrations promote lipolysis to provide adequate energy substrate for utilization by the body. However, chronically elevated cortisol concentrations, most commonly resulting from iatrogenic glucocorticoid administration to treat inflammatory diseases or, alternatively, because of ACTH‐ or cortisol‐secreting tumours, leads to weight gain and, in particular, accumulation of visceral adipose tissue.^3^ The reasons for these 2 apparently conflicting observations are unclear. Several studies have examined the effects of glucocorticoids on lipolysis; however, results have been inconsistent. For example, *in vitro* studies in adipocytes have shown lipolytic rates to be increased,^4^ unchanged^5^ or decreased^6^ by glucocorticoids. These discrepancies may be attributed to several factors, including the dose and duration of glucocorticoid treatment, the effect of other hormones in the media and the species being studied.

Results from *in vivo* studies testing the effect of glucocorticoids on lipolysis have been more consistent, particularly when levels of other counter‐regulatory hormones have been clamped by infusing somatostatin and replacing insulin, growth hormone and glucagon (the pancreatic clamp technique), showing that cortisol acutely increases whole body lipolysis.^7^, ^8^, ^9^ However, these studies achieved cortisol concentrations between 850 and 1500 nM which are not reflective of those observed physiologically in the absence of acute stress. Two studies have examined the effect of physiological cortisol concentrations on *in vivo* lipolysis and had conflicting results in the fasted state;^1^, ^10^ however, neither study used a pancreatic clamp to control counter‐regulatory hormones. Therefore, it is unclear whether changes in cortisol concentrations within the physiological range alter whole body lipolysis. It is also unclear how glucocorticoids enhance lipolysis in humans, as no *in vivo* study has examined the effect of glucocorticoids on the lipolytic pathway (Figure S1, Supporting Information).

Over and above prevailing glucocorticoid concentrations, a further critical confounder is the effect of other hormones regulating lipolysis, notably insulin and adrenaline.^5^, ^6^ *In vivo*, the effect of interactions between cortisol and either adrenaline^11^ or insulin^12^ has been examined only once in humans, although systemic rates of lipolysis were not measured as the appropriate tracers were not infused. We hypothesized that the effects of glucocorticoids on lipolysis in humans are indirect and dependent on the prevailing insulin and/or adrenaline concentrations, with glucocorticoids augmenting the pro‐lipolytic effects of adrenaline and antagonizing the suppressive effects of insulin. In addition, we hypothesized that these interactions may account for the apparently contradictory effects of acute (a state of high adrenaline and low insulin) and chronic (a state of high insulin and low adrenaline) glucocorticoid excess and that the effects may differ between subcutaneous and visceral depots. To test this, we performed a randomized, double‐blinded, crossover study to determine the effects of glucocorticoids on whole body lipolysis in the presence of both low and high insulin and adrenaline, respectively. Furthermore, we collected adipose tissue biopsies *in vivo* to determine how glucocorticoids alter lipolysis and tested *in vitro* whether glucocorticoids have differential effects on lipolysis in subcutaneous and visceral adipocytes.

## 2 MATERIALS AND METHODS

### 2.1 In vivo protocol

A total of 20 healthy men were recruited to a randomized, double‐blind, placebo‐controlled crossover study with the following inclusion criteria: age, 18 to 75 years; body mass index, 20 to 25 kg/m^2^; absence of chronic medical conditions; absence of regular medications; no previous glucocorticoid use in the past year; alcohol intake ≤21 units per week; weight change of <5% over the past 6 months; normal screening blood tests (renal, liver and thyroid function, fasting glucose, full blood count). Local ethical approval was obtained, as was written informed consent from each participant.

Subjects attended the Edinburgh Clinical Research Facility after overnight fast on 3 occasions, each separated by 3 weeks, and were instructed to avoid alcohol and exercise for 48 hours prior to each assessment. Volunteers were randomized to low, medium or high glucocorticoid (GC) phases (Figure S2, Supporting Information). The night prior to each assessment (11 pm) subjects received orally the 11β‐hydroxylase inhibitor metyrapone (metopirone) 1 g, along with either placebo (low GC phase), hydrocortisone 10 mg (medium GC) or hydrocortisone 20 mg (high GC). At 7 am the following morning subjects received orally 1 g of metyrapone along with either placebo (low GC), hydrocortisone 5 mg (medium GC) or hydrocortisone 10 mg (high GC). A further 1 g of metyrapone was received orally at 11 am to maintain inhibition of endogenous cortisol synthesis throughout the protocol. The 3 GC phases aimed to achieve trough and peak cortisol concentrations observed during normal diurnal rhythm (low and medium GC, respectively) and peak cortisol concentrations during stress (high GC).

**Figure 1:**
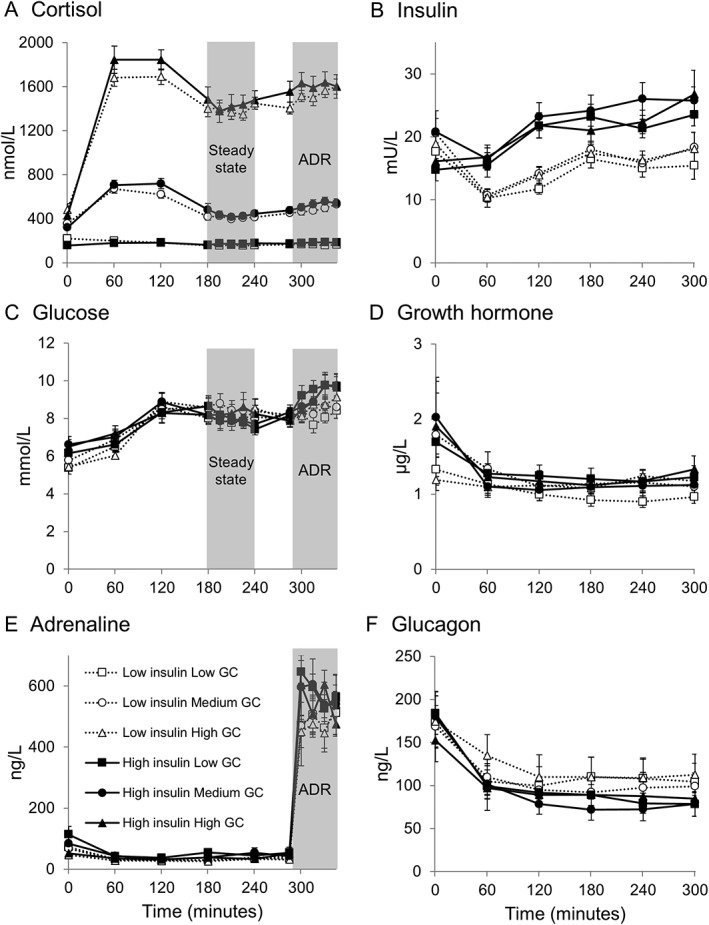
Hormone and metabolite concentrations during infusions. Data are given as mean ± SEM for n = 10 for low glucocorticoid (GC) (squares), medium GC (circles) and high GC (triangles) in low insulin (dotted lines, open shapes) and high insulin (solid lines, filled shapes) groups. A, Plasma cortisol was different between all 3 GC phases (P < .001). B, Serum insulin was increased in the high insulin group (P < .01) but unchanged by GC phase. C, Glucose; D, growth hormone; E, adrenaline; F, glucagon were unchanged by GC phase or between insulin groups. ADR, adrenaline infusion

At each visit, measurements were performed of height, weight, blood pressure, body fat by bioimpedance (using an Omron BF‐302 analyser) and core body temperature using a tympanic thermometer. Three cannulae were inserted (1 in a vein in each ante‐cubital fossa for infusions and 1 retrograde in a dorsal hand vein for arterialised sampling). Subjects placed their hand in a box heated to 60°C for 5 minutes prior to each arterialized sample collection. At t = 0 minutes, intravenous infusions of 6,6‐[^2^H]_2_‐glucose (at 0.22 µmol/kg/min following a 17.6 µmol/kg bolus) and 1,1,2,3,3‐[^2^H]_5_‐glycerol (at 0.11 µmol/kg/min following a 1.6 µmol/kg bolus) were commenced for 6.5 hours (Figure S2, Supporting Information). A “pancreatic clamp” was commenced at t = 0 minutes, comprising intravenous somatostatin (60 ng/kg/min), glucagon (0.5 ng/kg/min) and growth hormone (2 ng/kg/min). At their first visit, subjects were further randomized to receive either low or high insulin replacement (both groups n = 10) at a rate of 0.06 mU/kg/min or 0.2 mU/kg/min (aiming to suppress lipolysis by c. 50%),^13^ respectively, and subjects remained in this group for all 3 study visits. At t = 0 minutes, subjects commenced infusion with 0.9% saline (low GC) or hydrocortisone (medium GC at 0.025 mg/kg/h following a 0.04 mg/kg bolus; high GC at 0.12 mg/kg/h following a 0.18 mg/kg bolus) in random order. At t + 20 minutes, an intravenous infusion of 20% dextrose was commenced and the rate was adjusted every 10 minutes to maintain an arterialized glucose concentration between 7.5 and 8.0 mmol/L. Steady state measurements were taken between t + 180 and t + 240 minutes; following this a subcutaneous abdominal adipose tissue biopsy was obtained by needle aspiration.^14^ At t + 285 minutes, an adrenaline infusion was commenced at 0.15 nmol/kg/min for 60 minutes. Blood samples were obtained at regular intervals (Figure 1). Samples were stored at −80°C until analysis.

### 2.2 In vitro protocol

Paired samples of subcutaneous and visceral adipose tissue were obtained from patients undergoing elective abdominal surgery at the Royal Infirmary of Edinburgh (subject characteristics detailed in Table S1, Supporting Information). Adipose tissue from subcutaneous and visceral depots was digested, and the stromal vascular fraction was isolated and differentiated as previously described.^15^ In brief, following removal of connective tissue and blood vessels, adipose tissue was digested in collagenase type 1 (615 units/g tissue) for 90 minutes at 37°C. Following plating and overnight incubation in DMEM/F12 medium containing 33 μM biotin, 17 μM pantothenate and 10% foetal bovine serum, cells were differentiated for 3 days using serum‐free medium plus 1 nM triiodothyronine, 10 µg/mL transferrin, 66 nM insulin, 500 μM isobutylmethylxanthine, 1 μM dexamethasone and 10 μM rosiglitazone. From day 4 onwards, cells were maintained in differentiation medium, but without isobutylmethylxanthine, dexamethasone or rosiglitazone.

On day 16, cells were incubated with either 0, 100 or 1000 nM cortisol for 24 hours in the presence or absence of either vehicle, 100 pM insulin or 10 μM adrenaline. Following incubation, cells were used to measure mRNA levels of key genes in the lipolytic pathway or the medium was removed to measure the appearance of glycerol and cells were lysed and stored at −80°C for quantification of total protein.

### 2.3 Laboratory analyses

#### 2.3.1 Biochemical measurements

Serum lipids were measured on an Olympus Diagnostics analyser (County Clare, Ireland) using an enzymatic colorimetric method. Serum insulin, growth hormone, plasma glucagon and cortisol were measured by RIA kits (MP Biomedicals, Santa Ana, California). Serum non‐esterified fatty acids (NEFAs) were measured using a colorimetric assay (Wako Diagnostics, Mountain View, California) and plasma adrenaline by ELISA (Rocky Mountain Diagnostics, Colorado Springs, Colorado).

Endogenous and tracer glucose and glycerol concentrations *in vivo* were measured by GC‐MS as previously described.^16^ Glycerol in cell medium was measured in duplicate using a colorimetric kit (Sigma, Poole, UK). Protein in cell lysates was measured in duplicate using the DC protein assay (Bio‐Rad, Hercules, California) and glycerol appearance corrected for total protein.

#### 2.3.2 Quantitative real time PCR measurements

qPCR in whole adipose tissue and cultured adipocytes was performed as previously described.^17^ Primer sequences and probe numbers are described in Table S2, Supporting Information. Transcript levels are presented as the ratio of the abundance of the gene of interest: mean of abundance of control genes encoding cyclophilin A and 18S.

### 2.4 Kinetic analysis

Kinetic analysis for steady state (ss) measurements was performed using the mean of 5 samples obtained from t + 180 to t + 240 minutes. Steele's steady state equation^18^ was used to measure the rate of appearance (Ra) of glycerol as shown in Equation 1, where d5‐Glycerol TTR_ss_ is the tracer/tracee ratio (eg, d5‐Glycerol/Glycerol) during steady state:

(1) $$Ra\;\text{Glycero}l_{ss}\;=\frac{d5‐\text{Glycerol infusion rate}}{d5‐\text{Glycerol}\;TTR_{ss}}$$

The rate of disposal (Rd) of glucose was similarly calculated using Equation 2 while Ra glucose_ss_ was calculated by subtracting the mean of the glucose infusion rate (GIR_ss_) during steady state from Rd glucose_ss_:

(2) $$Rd\;\text{Glucos}e_{ss}\;=\frac{d2‐\text{Glucose}\;\text{infusion rate}}{d2‐\text{Glucose}\;TTR_{ss}}$$

(3) $$Ra\;\text{Glucos}e_{ss}\;=Rd\;\text{Glucos}e_{ss}-\;GIR_{ss}$$

Kinetic analysis following commencement of the adrenaline infusion was performed using Steele's modified non‐steady state equations.^18^ Ra glycerol and Rd glycerol were calculated as follows, where pV is volume of distribution:

(4) $$Ra\;\text{Glycero}l_{t2}=\frac{d5‐\text{Glycerol infusion rate}-pV\;\times \frac{\left[\text{Glycero}l_{t1}\right]+\left[\text{Glycero}l_{t2}\right]}{2}\times \frac{d5‐\mathrm{Glycerol}\;TTR_{t2}-d5‐\text{Glycerol}\;TTR_{t1}}{t_{2}-t_{1}}}{\frac{d5‐\text{Glycerol}\;TTR_{t1}+d5‐\text{Glycerol}\;TTR_{t2}}{2}}$$

(5) $$Rd\;\text{Glycero}l_{t2}=Ra\;\text{Glycero}l_{t2}-pV\times \frac{\left[\text{Glycero}l_{t2}\right]-\left[\text{Glycero}l_{t1}\right]}{t_{2}-t_{1}}$$

Non‐steady state values for Ra and Rd Glucose were calculated as above, substituting glucose for glycerol, with the addition that the GIR was subtracted from the total Ra glucose to determine the endogenous glucose production as in Equation 3. The effective volume of distribution (pV) used for glycerol was 230 mL/kg.^19^, ^20^ For glucose, different values were tested for the pV which comprised 40, 100 and 150 mL/kg.^19^ The results were not significantly altered by any of these pV values, probably because d2‐glucose enrichment was not significantly altered by adrenaline infusion. The results presented for glucose kinetics are those using 100 mL/kg as the pV.

### 2.5 Statistical analysis

Data are presented as mean ± SEM. SPSS version 19 was used for all analyses. Comparisons between phases (ie, effect of glucocorticoids during steady state) were tested by 2‐way repeated measures ANOVA with post‐hoc testing performed using Fisher's least squares differences (LSD) test with the effect of insulin as an independent variable. Comparisons over time (ie, effect of adrenaline infusion) were tested by 2‐way repeated measures ANOVA with post‐hoc LSD testing, with effects of insulin and glucocorticoids as independent variables. *P* < .05 was considered significant.

## 3 RESULTS

### 3.1 Regulation of lipolysis by glucocorticoids in vivo

Subject characteristics are shown in Table 1. Subjects in the low and high insulin groups were of similar age, weight and blood pressure and had similar biochemical measurements.

**Table 1:** Anthropometric and fasting biochemical measurements

	Low insulin (n = 10)			High insulin (n = 10)		
	Low GC	Medium GC	High GC	Low GC	Medium GC	High GC
Age (years)	36.9 ± 4.7	37.0 ± 4.7	36.9 ± 4.7	29.9 ± 5.2	30.0 ± 5.2	29.9 ± 5.2
Weight (kg)	74.6 ± 2.1	75.3 ± 2.0	75.1 ± 2.3	76.6 ± 2.8	76.9 ± 2.9	76.7 ± 2.7
BMI (kg/m^2^)	23.2 ± 0.5	23.5 ± 0.5	23.4 ± 0.6	24.0 ± 0.4	24.1 ± 0.5	24.1 ± 0.5
Fat mass (kg)	13.6 ± 0.8	14.1 ± 1.0	14.1 ± 0.9	12.7 ± 1.2	13.4 ± 1.3	13.0 ± 1.3
Temperature (°C)	36.6 ± 0.2	36.6 ± 0.1	36.5 ± 0.2	36.6 ± 0.1	36.7 ± 0.1	36.7 ± 0.1
Cholesterol (mmol/L)	3.9 ± 0.2	4.3 ± 0.1	4.1 ± 0.2	4.2 ± 0.3	4.2 ± 0.3	4.3 ± 0.2
Triglycerides (mmol/L)	0.9 ± 0.1	0.9 ± 0.1	0.9 ± 0.1	1.0 ± 0.1	1.0 ± 0.2	0.8 ± 0.1
NEFAs (µmol/L)	286 ± 37	288 ± 31	341 ± 38	317 ± 40	292 ± 53	254 ± 42
Heart rate (bpm) (SS)	61.0 ± 2.9	62.7 ± 2.8	59.7 ± 2.1	59.4 ± 2.8	59.2 ± 2.3	61.4 ± 2.0
Heart rate (bpm) (ADR)	66.4 ± 2.3	69.6 ± 3.0	76.1 ± 1.9*#	67.5 ± 2.3	68.7 ± 3.2	75.7 ± 2.7**##
Systolic BP (mm Hg) (SS)	120.8 ± 2.1	122.1 ± 2.0	123.4 ± 2.4	119.1 ± 2.7	120.5 ± 2.2	124.4 ± 2.5
Systolic BP (mm Hg) (ADR)	126.4 ± 2.7	127.3 ± 2.9	131.3 ± 3.6	127.6 ± 3.2	131.1 ± 2.7	130.7 ± 5.2
Diastolic BP (mm Hg)(SS)	72.1 ± 2.5	74.2 ± 2.2	73.4 ± 1.9	68.8 ± 1.9	69.4 ± 1.9	69.8 ± 1.4
Diastolic BP (mm Hg) (ADR)	69.7 ± 2.2	67.3 ± 2.5	65.7 ± 2.1	63.0 ± 1.7	65.3 ± 2.0	61.6 ± 2.5
Total glucose infused (g)	21.4 ± 2.8	12.7 ± 1.9*	14.9 ± 2.3*	62.5 ± 6.5	54.5 ± 7.6	53.8 ± 6.8

Data are given as mean ± SEM. Steady state (SS) data are the mean values obtained from t + 180 to t + 240 minutes of the infusion. Adrenaline (ADR) data are the mean values obtained from t + 285 to t + 345 minutes. Adrenaline increased heart rate and systolic BP and decreased diastolic BP (all P < .01). Total glucose infused during the protocol was increased in the high insulin group (P < .001). NEFAs = non‐esterified fatty acids. *P < .05, **P < .01 vs low GC; #P < .05, ##P < .01 vs medium GC.

#### 3.1.1 Baseline measurements at study visits

Cortisol concentrations were different between GC phases (all *P* < .01) (Figure 1A). Fasting insulin, glucose, growth hormone, glucagon and adrenaline (Figure 1), NEFAs and glycerol (data not shown) were similar between insulin groups and unaltered by GC phase.

#### 3.1.2 Steady state measurements

Cortisol concentrations remained different between phases (all *P* < .001) and were similar between high and low insulin groups (Figure 1A). Insulin concentrations were increased in the high insulin group (*P* < .01) (Figure 1B). Glucose, growth hormone, glucagon and adrenaline concentrations were similar between GC phases and insulin groups (Figure 1C‐F). Concentrations of these hormones and glucose remained stable during steady state. High GC tended to increase systolic blood pressure (*P* = .06, Table 1).

##### 3.1.2.1 Effects of glucocorticoids on lipolysis and glucose kinetics

**Figure 2:**
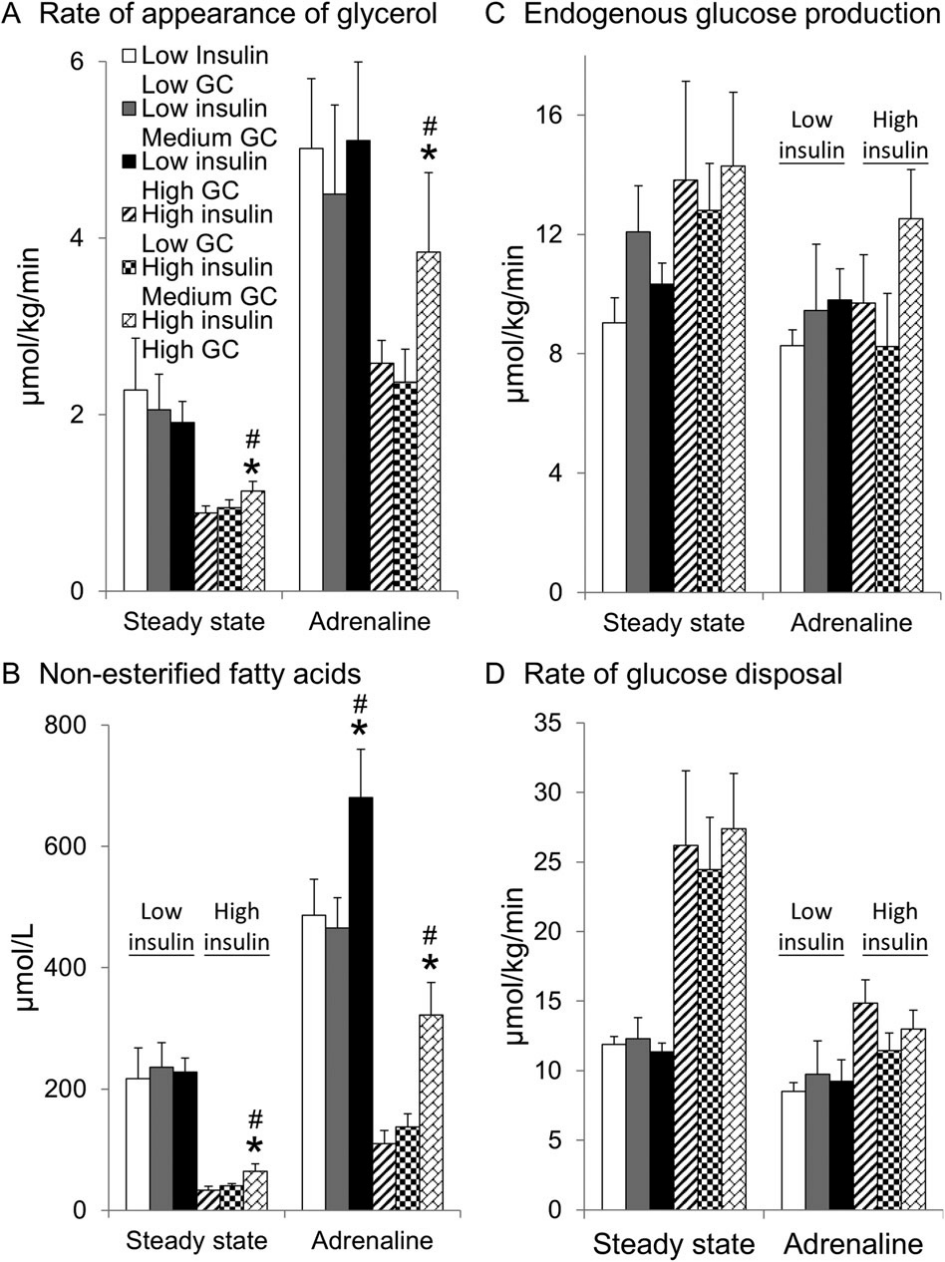
Effect of glucocorticoids on lipolysis and glucose kinetics in vivo. Data are given as mean ± SEM for n = 10 for low insulin (comprising low glucocorticoid [GC] phase [white columns], medium GC [grey columns] and high GC [black columns]) and high insulin (low GC [striped columns], medium GC [checked columns] and high GC [bricked columns]) groups. A, High GC increased the rate of appearance of glycerol in the high insulin group at steady state (t + 180‐240 minutes) and during adrenaline infusion (t + 285‐345 minutes). B, High GC increased NEFA concentrations in the high insulin group during steady state and in both insulin groups during adrenaline infusion. High insulin suppressed the rate of appearance of glycerol and NEFAs (both P < .001). GC phase did not alter either C, endogenous glucose production or D, glucose disposal. Glucose disposal was increased in the high insulin group during steady state (P < .01) but not during adrenaline infusion. Adrenaline decreased glucose disposal in both groups (P < .01). *P < .05 vs low GC, #P < .05 vs medium GC

Ra glycerol and NEFA concentrations were suppressed by high insulin (Figure 2A,B). The high GC phase increased Ra glycerol and NEFAs only in the high insulin group (Figure 2A,B).

The high insulin group required more intravenous glucose to maintain glucose concentrations over the 345‐minute protocol (Table 1). In the low insulin group, the medium and high GC phases reduced the required glucose infusion rate (Table 1). Endogenous glucose production (EGP) and Rd glucose were unchanged by GC phase in either insulin group (Figure 2C,D).

#### 3.1.3 Measurements during adrenaline infusion

Adrenaline concentrations achieved during the infusion were similar to those observed during exercise^21^ (Figure 1E). Adrenaline increased heart rate and systolic blood pressure and decreased diastolic blood pressure (Table 1). The high GC phase increased heart rate during the adrenaline infusion in both insulin groups (Table 1).

##### 3.1.3.1 Effects of glucocorticoids on lipolysis and glucose kinetics

Adrenaline increased Ra glycerol and NEFAs (all phases *P* < .001) (Figure 2A,B). High GC increased Ra glycerol and NEFAs in the high insulin group (Figure 2A,B). In contrast, high GC did not increase Ra glycerol in the low insulin group, although NEFAs were increased (Figure 2A,B). GC phase did not alter Rd glycerol (data not shown).

Adrenaline increased glucose concentrations only in the high insulin group (*P* < .05) (Figure 1C) and decreased Rd glucose in both insulin groups (*P* < .01) (Figure 2D). GC phase did not alter EGP or Rd glucose in either insulin group (Figure 2C,D).

#### 3.1.4 Glucocorticoids increase expression of key genes in the lipolytic pathway

**Figure 3:**
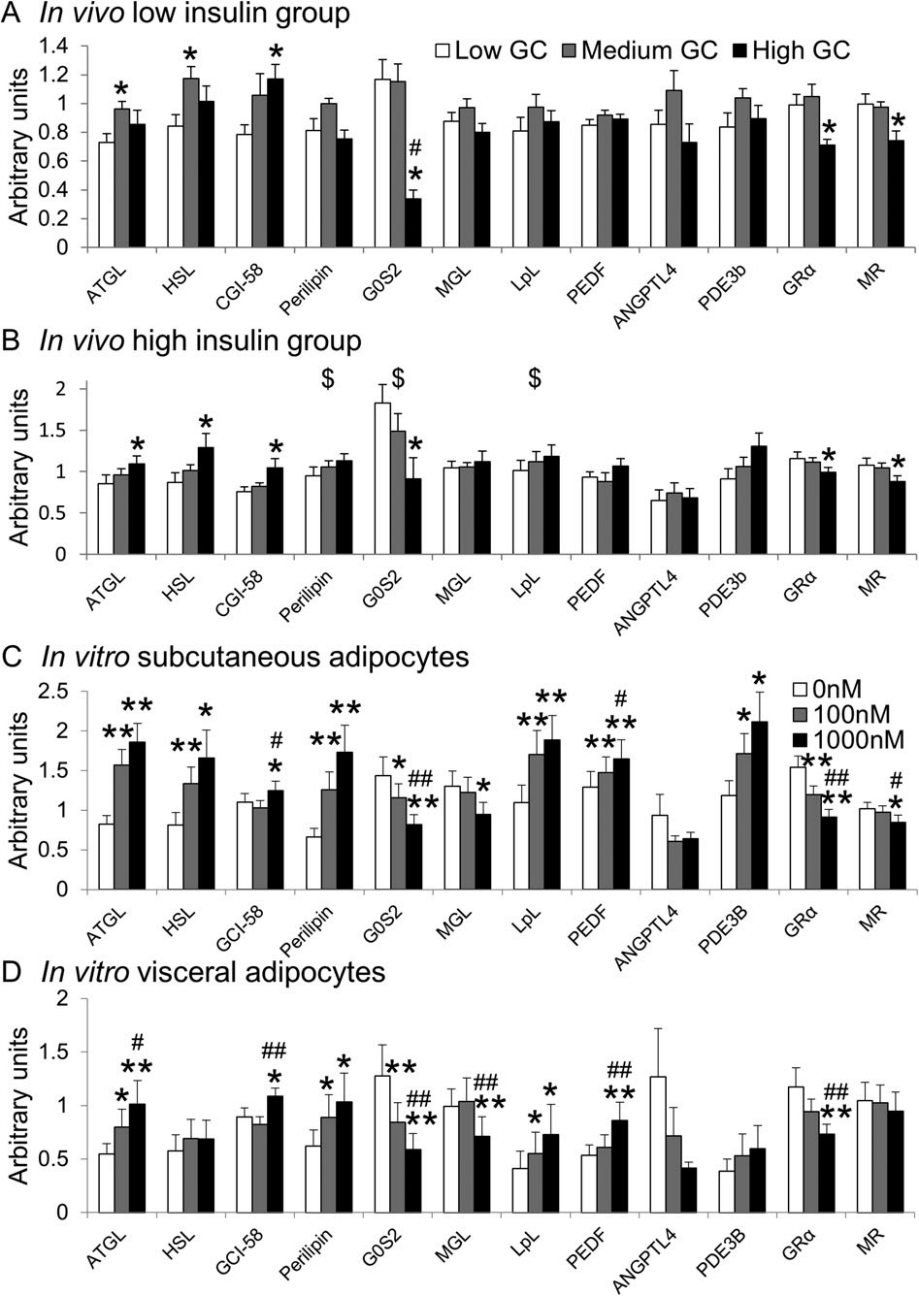
Glucocorticoid regulation of adipose mRNA levels in vivo and in vitro. A, B, Data are given as mean ± SEM for low glucocorticoid (GC) (white columns), medium GC (grey columns) and high GC (black columns) for A, low insulin (n = 10) and B, high insulin (n = 9) groups. Medium or high GC increased mRNA levels of ATGL, HSL and CGI‐58 and suppressed G0S2. High insulin increased perilipin‐1, G0S2 and LpL mRNA levels. C,D, Data are given as mean ± SEM for C, subcutaneous and D, visceral adipocytes (both n=11) cultured for 24 hours in 0 nM (white columns), 100 nM (grey columns) or 1000 nM cortisol (black columns). While in vitro regulation of transcripts by cortisol was similar to in vivo data, cortisol also increased perilipin‐1, LpL, PEDF, PDE3b and decreased MGL levels in vitro. HSL was increased by cortisol only in the subcutaneous adipocytes. *P < .05, **P < .01 vs low GC/0 nM; #P < .05, ##P < .01 vs medium GC/100 nM. $P < .05 vs low insulin group

Adipose tissue was analysed in low (n = 10) and high (n = 9) insulin groups. Adipose tissue from one subject in the high insulin group was not obtained because of technical difficulties with the biopsy procedure. The high GC phase increased mRNA levels of key lipolytic genes adipose triglyceride lipase (ATGL), hormone sensitive lipase (HSL) and comparative gene identification‐58 (CGI‐58) and decreased G0/G1 switch 2 (*G0S2*) (Figure 3A,B). In addition, high GC decreased transcript levels of the glucocorticoid (GRα) and mineralocorticoid receptor (MR). Despite the GC phase not increasing lipolysis in the low insulin group, GC similarly regulated the lipolytic pathway in both insulin groups (Figure 3A,B). Perilipin 1, *G0S2* and lipoprotein lipase were increased by high insulin (Figure 3B).

### 3.2 Regulation of lipolysis by glucocorticoids in vitro

**Figure 4:**
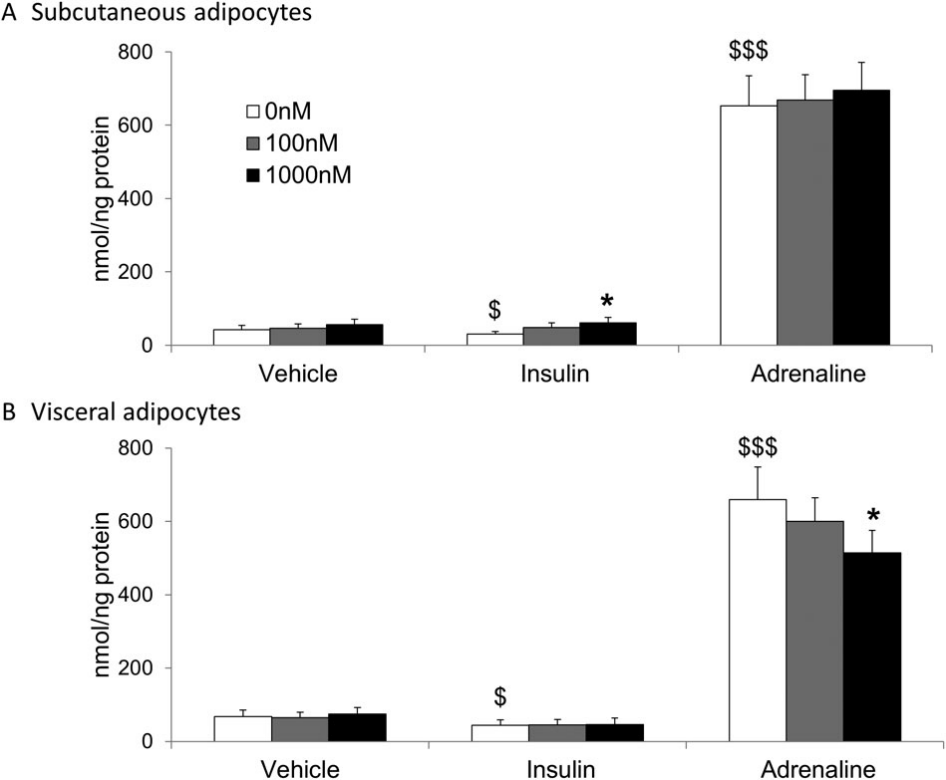
Glucocorticoid regulation of lipolysis in vitro. Data are given as mean ± SEM for glycerol release from paired primary human subcutaneous (A) and visceral adipocytes (B) (both n=11) cultured in 0 nM (white columns), 100 nM (grey columns) or 1000 nM cortisol (black columns) for 24 hours in the presence of either vehicle, 100 pM insulin or 10 µM adrenaline. Insulin suppressed and adrenaline increased glycerol release. Cortisol did not alter glycerol release from adipocytes in vehicle treated cells, but increased glycerol release in subcutaneous adipocytes co‐incubated with insulin. Cortisol decreased glycerol release in visceral adipocytes co‐incubated with adrenaline. *P < .05 vs 0 nM; $P < .05, $$$P < .001 vs vehicle 0 nM

Cortisol did not increase glycerol appearance (a measure of lipolysis) during incubations without insulin or adrenaline in subcutaneous or visceral differentiated pre‐adipocytes in agreement with *in vivo* data (Figure 4). Insulin suppressed and adrenaline increased glycerol appearance in both subcutaneous and visceral adipocytes. In the presence of insulin, cortisol increased glycerol appearance in subcutaneous but not visceral adipocytes (Figure 4). In the presence of adrenaline, cortisol suppressed glycerol appearance in visceral adipocytes.

As *in vivo*, cortisol increased mRNA levels of ATGL, HSL and CGI‐58 and suppressed *G0S2*, GRα and MR in subcutaneous adipocytes (Figure 3C). Similar results were observed in visceral adipocytes; however, cortisol did not alter HSL (Figure 3D). In contrast with *in vivo* data, cortisol increased mRNA levels of perilipin‐1, *LPL*, PEDF and *PDE3B* and suppressed MGL.

## 4 DISCUSSION

This work shows that glucocorticoids are dependent on insulin and/or adrenaline in order to increase whole body lipolysis *in vivo*. In the presence of low insulin levels (c. 16 mU/L), even circulating cortisol concentrations of c. 1400 nM did not increase Ra glycerol or NEFA concentrations. Conversely, in slightly higher insulin concentrations (c. 22 mU/L) designed to suppress lipolysis by 50%, high cortisol concentrations increased Ra glycerol by 20% to 25%, showing that cortisol antagonizes the effect of insulin. In addition, high cortisol augmented the pro‐lipolytic effects of adrenaline in the high insulin group. However, high GC did not enhance Ra glycerol in the low insulin group during adrenaline infusion, but did increase circulating NEFAs. Therefore, our study provides the first *in vivo* evidence that glucocorticoids have permissive effects on lipolysis in humans. Although our data might appear to contrast with some previous work,^7^, ^8^, ^9^ in one of those studies replacement glucagon was not infused, while we infused a lower insulin dose than the other 2 studies and, as we have shown, a “threshold” insulin dose is required to mediate the lipolytic effects of cortisol. In addition, unlike the other studies, we used metyrapone to control cortisol levels during all phases and performed longer treatment with hydrocortisone. However, metyrapone does not alter lipolysis independently of its effects on cortisol,^10^ while cortisol accumulates slowly in adipose tissue, meaning that a longer duration would be more likely to identify effects on lipolysis.^22^

Although previous studies have tested how glucocorticoids regulate the lipolytic pathway in rodents (by increasing ATGL and HSL),^4^, ^23^ this had not been tested *in vivo* in humans. Glucocorticoids act predominantly by altering transcription of target genes and we found that cortisol increased transcription of the key lipases ATGL (and the co‐factor CGI‐58) and HSL and suppressed the negative lipolytic regulator *G0S2*. Interestingly, this regulation was similar in both insulin groups, despite cortisol not enhancing lipolysis during low insulin. This shows that glucocorticoids “prime” the pathway, to enhance lipolysis in response to adrenaline or to antagonize the effects of insulin. Additional genes outside the classic lipolytic pathway have been implicated in the lipolytic effects of glucocorticoids, such as phosphodiesterase 3b,^4^ pigment epithelium derived factor^24^ and angiopoietin‐like 4;^25^ however, in our study, cortisol did not alter mRNA levels of these genes *in vivo,* while *in vitro* PDE3b levels were in fact increased, suggesting that glucocorticoids do not antagonize the effect of insulin via this pathway in humans. There are additional mechanisms through which glucocorticoids might promote or inhibit the effects of adrenaline and insulin which we did not explore; for example, glucocorticoids increase the number of pro‐lipolytic β‐receptors on the adipocyte membrane,^26^, ^27^ decrease the number of anti‐lipolytic α2‐receptors^28^ and increase cAMP levels in rodent adipocytes.^4^ However, glucocorticoids enhance basal lipolysis in rodents,^4^, ^23^ which we did not observe in humans, highlighting species‐specific differences in the glucocorticoid regulation of lipolysis. Interestingly, high insulin increased mRNA levels of perilipin 1, *G0S2* 29 and lipoprotein lipase, which should all reduce lipolysis and/or promote lipogenesis, in agreement with the known effects of insulin.

Subcutaneous and visceral adipocytes arise from different cell lineages,^30^ and we tested whether glucocorticoids have depot‐specific effects in paired subcutaneous and visceral adipocytes. Importantly, insulin suppressed and adrenaline stimulated glycerol release similarly in both cell types. In agreement with *in vivo* data, cortisol increased lipolysis only in the presence of insulin in the subcutaneous adipocytes and did not increase lipolysis in the presence of adrenaline without insulin. However, cortisol failed to enhance lipolysis in the visceral adipocytes even with insulin and decreased lipolysis in the presence of adrenaline. Although visceral adipose tissue accounts for only a small proportion (c. 6%) of whole body lipolysis in lean individuals,^31^ this may explain why chronic glucocorticoid excess causes accumulation of visceral adipose tissue. Although GRα and MR transcript levels were similar between depots, cortisol failed to increase HSL levels in visceral adipocytes, which could be responsible, in part, for these depot‐specific differences, as catecholamines and insulin both mediate their effects on lipolysis by phosphorylating HSL. Although we tested only the acute effects of cortisol on lipolysis, chronic glucocorticoid excess causes hyperinsulinaemia, which would suppress lipolysis and promote lipogenesis. The high GC phase only partially antagonized the insulin‐mediated suppression of lipolysis; thus, cortisol‐induced hyperinsulinaemia may offset this effect, which may explain why chronic GC excess does not necessarily increase whole‐body lipolysis.^32^, ^33^

It is of note that only supraphysiological cortisol concentrations increased lipolysis while cortisol levels similar to physiological early morning cortisol concentrations (c. 400 nM) did not increase whole‐body lipolysis compared with low cortisol (c.150 nM). This suggests that acute diurnal variation in cortisol concentrations may not substantially alter lipolysis during normal physiology, although the physiological response to “clamped” cortisol levels may differ from ultradian rhythm.^34^ In addition, we clamped several other hormones to prevent confounding effects, and the physiological rise in these hormones may be critical for the glucocorticoid‐dependent diurnal variation in lipolysis.^10^ In addition, it is possible that cortisol concentrations less than 150 nM (eg, in Addison's disease) would have reduced lipolysis further, although *in vitro* 0 nM cortisol did not reduce lipolysis compared with 100 nM. Another unexpected finding was the absence of effect of even high cortisol concentrations on glucose uptake or production, highlighting the fact that the known effects of glucocorticoids to raise blood glucose concentrations are also permissive and are probably mediated through effects on other tissues such as the pancreas. However, the glucose infusion rate was reduced on low GC compared with the other phases in the low‐insulin group, and it is possible that, with greater numbers, we would have found a reduction in endogenous glucose production. Furthermore, cortisol may have enhanced glucose uptake and/or suppressed glucose disposal if we had infused larger doses of insulin. This protocol, however, was designed to suppress lipolysis by 50% and not to substantially alter glucose kinetics.

To conclude, the acute lipolytic effects of glucocorticoids are dependent on insulin and adrenaline and are observed in subcutaneous, but not visceral, adipose tissue solely during supraphysiological cortisol concentrations. These effects are mediated, at least in part, by enhanced transcription of ATGL, HSL and CGI‐58 and by suppression of *G0S2*. These findings highlight how the permissive lipolytic effects of glucocorticoids are probably mediated and indicate the importance of hormonal interactions in regulating energy balance.

## Supporting information


**Table** **S1** **.** Participants for in vitro experiment.
**Table** **S2** **.** Primer sequences for qPCR and corresponding probe numbers. All assays were performed using the following primer probe sequences and the Roche probe library, except for hormone sensitive lipase which was measured using the SYBR green assay.
**Figure** **S1** **.** The lipolytic pathway. Schematic representing the major steps in the lipolytic pathway in a lipid droplet of an adipocyte. Triacylglycerides (TAG) are converted to diacylglycerides (DAG) with release of a fatty acid (FA) by adipose triglyceride lipase (ATGL) along with ATGL’s co‐factor comparative gene identification‐58 (CGI‐58). ATGL function is inhibited by G0/G1 switch 2 (G0S2). DAGs are converted to monoacylglycerides (MAG) with FA release by hormone sensitive lipase (HSL) following interaction with perilipin‐1 (PLIN‐1). Finally, MAGs are hydrolysed by monoglyceride lipase (MGL) with release of glycerol and FA.
**Figure** **S2** **.** Study protocol. A, Flowchart depicting study design, with 20 men initially randomised to either low or high insulin groups (both n = 10). Subjects attended the Clinical Research Facility on three occasions in random order and received metyrapone +/− hydrocortisone the evening before each study visit at 23:00 and at 07:00 hours on the morning of their assessment to ensure glucocorticoid (GC) levels were either low, medium or high. B, Protocol during each study visit. Infusions were commenced at the times shown and steady state blood samples were taken between t + 180 and t + 240 minutes. An adipose tissue (AT) biopsy was performed at t + 240 minutes prior to commencement of an adrenaline infusion for the final hour of the protocol.
