# Supplementary material for: Acute physiological effects of glucocorticoids on fuel metabolism in humans are permissive but not direct
Source: Diabetes Obes Metab. 2017 Mar 28;19(6):883–91. doi: 10.1111/dom.12899 (PMC5484992; doi:10.1111/dom.12899)

**Supplementary Information**

***Table S1 Participants for in vitro experiment***

| **Subject number** | **Operation** | **Age (y)** | **Sex** | **BMI (kg/m^2^)** |
| --- | --- | --- | --- | --- |
| **1** | Cholecystectomy | 66 | Male | 27.2 |
| **2** | Abdominal hernia repair | 70 | Female | 24.9 |
| **3** | Cholecystectomy and open hernia repair | 53 | Female | 42.5 |
| **4** | Abdominal hernia repair | 68 | Female | 27.6 |
| **5** | Abdominal hernia repair | 73 | Male | 27.5 |
| **6** | Abdominal hernia repair | 66 | Female | 35.2 |
| **7** | Abdominal hernia repair | 49 | Female | 43.5 |
| **8** | Abdominal hernia repair | 69 | Male | 30.8 |
| **9** | Abdominal hernia repair | 68 | Male | 32.7 |
| **10** | Abdominal hernia repair | 36 | Female | 41.3 |
| **11** | Abdominal hernia repair | 66 | Male | 31.7 |

***Table S2 Primer sequences for qPCR and corresponding probe numbers***

*All assays were performed using the following primer probe sequences and the Roche probe library, except for hormone sensitive lipase which was measured using the SYBR green assay.*

| Gene Name | Primer sequences 5’ to 3’ | Roche UPL Probe number |
| --- | --- | --- |
| *PPIA*  (cyclophilin A) | F: atgctggacccaacacaat | 48 |
|  | R: tctttcactttgccaaacacc |  |
| *RNA18S5*  (18S) | F: cttccacaggaggcctacac | 46 |
|  | R: cgcaaaatatgctggaacttt |  |
| *PNPLA2*  (ATGL) | F: ctccaccaacatccacgag | 89 |
|  | R: ccctgcttgcacatctctc |  |
| *LIPE*  (hormone sensitive lipase) | F: ggaagtgctatcgtctctgg | SYBR green |
|  | R: ggcagtcagtggcatctc |  |
| *ABHD5*  (CGI-58) | F: ggacaaaatgatcttgcttgg | 66 |
|  | R: cccaaggctccactaaaatg |  |
| *G0S2*  (G0/G1 switch 2) | F: ggaggagaacgctgaggtc | 15 |
|  | R: tttccatctcggctctgg |  |
| *PLIN1*  (perilipin 1) | F: aggatggcagtcaacaaagg | 42 |
|  | R: gcagcacattctcctgctc |  |
| *MGLL*  (monoglyceride lipase) | F: cgtgctctctcggaataagac | 41 |
|  | R: agttggatgccgaagcac |  |
| *LPL*  (lipoprotein lipase) | F: atgtggcccggtttatca | 25 |
|  | R: ctgtatcccaagagatggacatt |  |
| *SERPINF1*  (pigment epithelium derived factor) | F: gtgtggagctgcagcgtat | 57 |
|  | R: tccaatgcagaggagtagca |  |
| *ANGPTL4*  (angiopoietin like 4) | F: gtggaccctgaggtccttc | 18 |
|  | R: ccaccttgtggaagagttgc |  |
| *PDE3B*  (phosphodiesterase 3b) | F: gagaaaggggatagaaaacttaacaa | 73 |
|  | R: gtagcaatcctgaagttcctgag |  |
| *NR3C1*  (glucocorticoid receptor-α) | F: ttttcttcaaaagagcagtgga | 11 |
|  | R: gcatgctgggcagttttt |  |
| *NR3C2*  (mineralocorticoid receptor) | F: catcatgaaagttttgctgctact | 64 |
|  | R: tctttgatgtaatttgtcctcatttc |  |

**Figure legends**

***Figure S1 The lipolytic pathway***

Schematic representing the major steps in the lipolytic pathway in a lipid droplet of an adipocyte. Triacylglycerides (TAG) are converted to diacylglycerides (DAG) with release of a fatty acid (FA) by adipose triglyceride lipase (ATGL) along with ATGL’s co-factor comparative gene identification-58 (CGI-58). ATGL function is inhibited by G0/G1 switch 2 (G0S2). DAGs are converted to monoacylglycerides (MAG) with FA release by hormone sensitive lipase (HSL) following interaction with perilipin-1 (PLIN-1). Finally, MAGs are hydrolysed by monoglyceride lipase (MGL) with release of glycerol and FA.

***
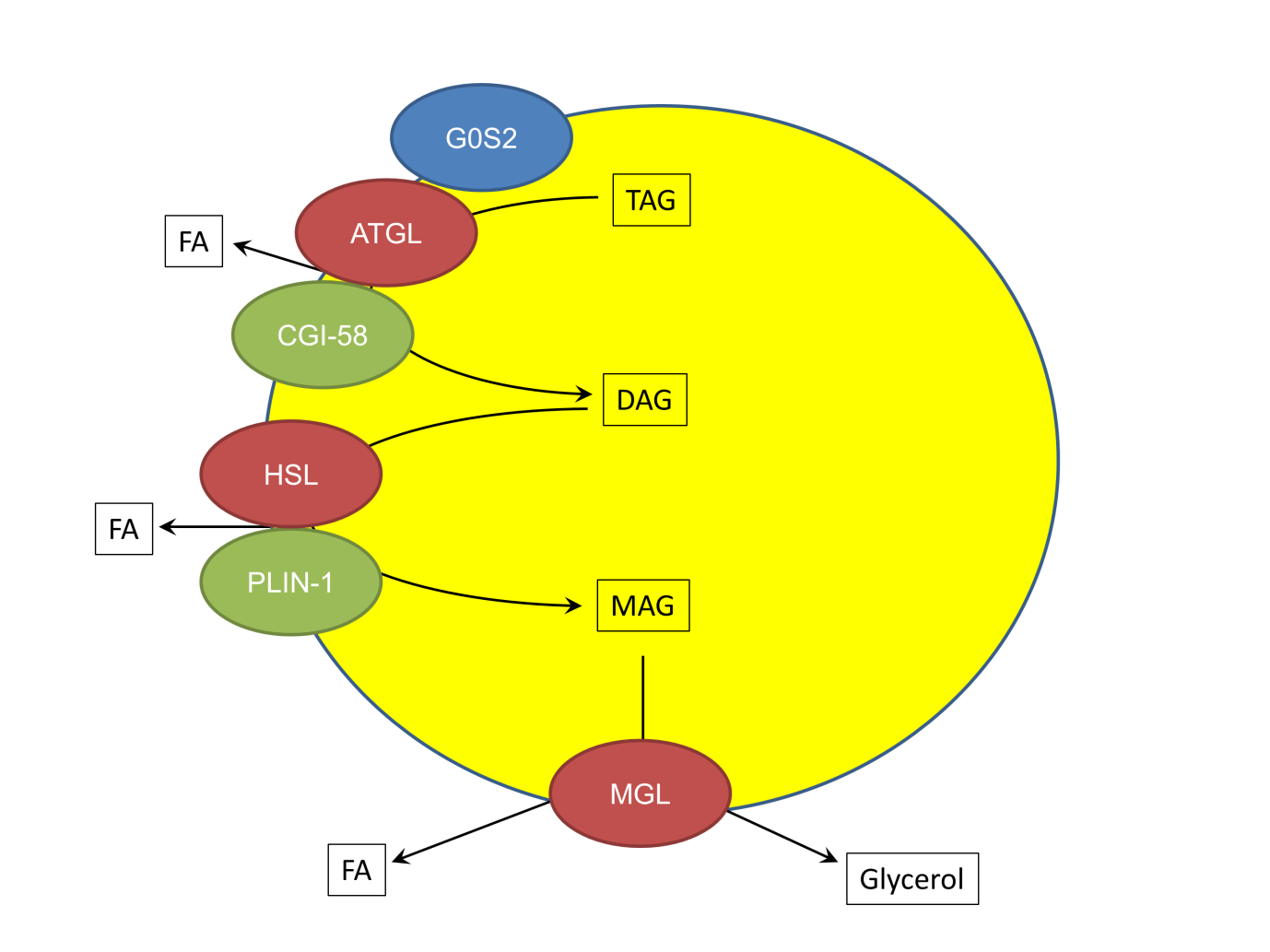
***

***Figure S2 Study protocol***

*A) Flowchart depicting study design, with 20 men initially randomised to either low or high insulin groups (both n=10). Subjects attended the Clinical Research Facility on three occasions in random order and received metyrapone +/- hydrocortisone the evening before each study visit at 2300h and at 0700h on the morning of their assessment to ensure glucocorticoid (GC) levels were either low, medium or high. B) Protocol during each study visit. Infusions were commenced at the times shown and steady state blood samples were taken between t+180 and t+240 minutes. An adipose tissue (AT) biopsy was performed at t+240 minutes prior to commencement of an adrenaline infusion for the final hour of the protocol.*


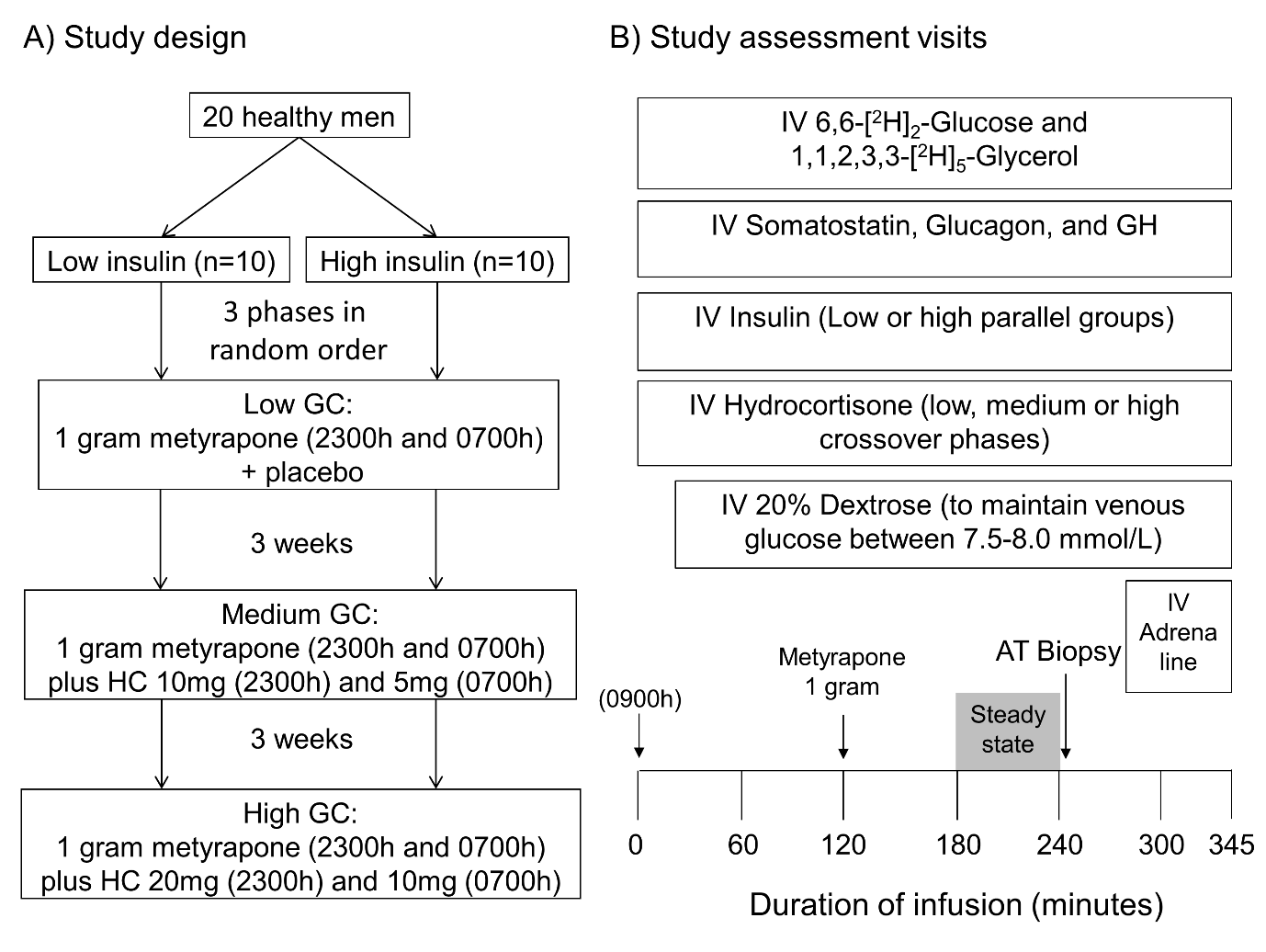

Supplement: Supplementary file 1 — Table S1 . Participants for in vitro experiment. Table S2 . Primer sequences for qPCR and corresponding probe numbers. All assays were performed using the following primer probe sequences and the Roche probe library, except for hormone sensitive lipase which was measured using the SYBR green assay. Figure S1 . The lipolytic pathway. Schematic representing the major steps in the lipolytic pathway in a lipid droplet of an adipocyte. Triacylglycerides (TAG) are converted to diacylglycerides (DAG) with release of a fatty acid (FA) by adipose triglyceride lipase (ATGL) along with ATGL’s co‐factor comparative gene identification‐58 (CGI‐58). ATGL function is inhibited by G0/G1 switch 2 (G0S2). DAGs are converted to monoacylglycerides (MAG) with FA release by hormone sensitive lipase (HSL) following interaction with perilipin‐1 (PLIN‐1). Finally, MAGs are hydrolysed by monoglyceride lipase (MGL) with release of glycerol and FA. Figure S2 . Study protocol. A, Flowchart depicting study design, with 20 men initially randomised to either low or high insulin groups (both n = 10). Subjects attended the Clinical Research Facility on three occasions in random order and received metyrapone +/− hydrocortisone the evening before each study visit at 23:00 and at 07:00 hours on the morning of their assessment to ensure glucocorticoid (GC) levels were either low, medium or high. B, Protocol during each study visit. Infusions were commenced at the times shown and steady state blood samples were taken between t + 180 and t + 240 minutes. An adipose tissue (AT) biopsy was performed at t + 240 minutes prior to commencement of an adrenaline infusion for the final hour of the protocol. [file DOM-19-883-s001.docx]
